# Structural and Functional Diversity among Five RING Finger Proteins from Carassius Auratus Herpesvirus (CaHV)

**DOI:** 10.3390/v13020254

**Published:** 2021-02-07

**Authors:** Zi-Hao Wang, Fei Ke, Qi-Ya Zhang, Jian-Fang Gui

**Affiliations:** 1 State Key Laboratory of Freshwater Ecology and Biotechnology, Institute of Hydrobiology, Chinese Academy of Sciences, Wuhan 430072, China; wangzh@ihb.ac.cn (Z.-H.W.); kefei@ihb.ac.cn (F.K.); 2 College of Modern Agriculture Sciences, University of Chinese Academy of Sciences, Beijing 100049, China; 3 The Innovation Academy of Seed Design, Chinese Academy of Sciences, Beijing 100101, China

**Keywords:** *Carassius auratus* herpesvirus (CaHV), Fish herpesvirus, RING finger proteins, structural diversity, ubiquitination activity, subcellular localization, virus-host interaction

## Abstract

*Carassius auratus* herpesvirus (CaHV) has been identified as a high-virulence pathogenic virus that infects aquatic animals, but the key factor for virus–host interaction is still unclear. Five Really interesting new genes (RING) finger proteins (39L, 52L, 131R, 136L, and 143R) of CaHV were screened to determine structural diversity. RING finger proteins were also predicted in other known fish herpesviruses, with an arrangement and number similar to CaHV. We performed multifaceted analyses of the proteins, including protein sizes, skeleton structures, subcellular localizations, and ubiquitination activities, to determine their precise roles in virus–host interactions. The five proteins were overexpressed and detected different levels of ubiquitination activities, and 143R showed the highest activity. Then, the prokaryotic expressed and purified full-length proteins (131R and 136L), RING domain isolates (131R_12–43_ and 136L_45–87_), and RING domain-deleted mutants (131RΔ_12–43_ and 136LΔ_45–87_) were prepared to detect their activities through ubiquitination assays. The results indicate that both full-length proteins and their isolates have activities that catalyze ubiquitination, and the full-length proteins possess higher activity than the isolates, but RING domain-deleted mutants lose their activities. Furthermore, the activities of the five proteins were verified as E3 ubiquitin ligase activity, showing that the RING domains determine the ubiquitination activity. These proteins present different subcellular localization. RING domain-deleted mutants showed similar subcellular localization with their full-length proteins, and all the isolates diffused in whole cells. The current results indicate that the sequence outside the RING domain determines subcellular localization and the level of ubiquitination activity, suggesting that the RING finger proteins of fish herpesviruses might have diverse functions in virus–host interaction.

## 1. Introduction

The rapid development of the aquaculture industry has made outstanding contributions to protecting global food nutrition and safety [1,2]. Aquaculture is recognized as a highly efficient system for producing protein for human consumption, including the important economic fish crucian carp (*Carassius auratus*), with an annual output of nearly three million tons in China [3,4,5]; however, the prevalence of viral diseases, especially herpesvirus diseases, reported to be widespread in aquatic animals [6,7], has seriously affected the production and quality of aquatic products [8]. *Carassius auratus* herpesvirus (CaHV), isolated from diseased crucian carp, is a member of the genus *Cyprinivirus* within the family *Alloherpesviridae* in the order *Herpesvirales* that includes cyprinid herpesvirus 1 (CyHV-1), cyprinid herpesvirus 2 (CyHV-2), and cyprinid herpesvirus 3 (CyHV-3) [9,10,11,12]. A genome sequence was completed, and genome architecture, some open reading frames’ (ORFs) insertion or deletion, and nucleotide sequence were found to be different from other members of the genus *Cyprinivirus* [13]. Several studies have also been carried out on the viral genes, subcellular localization of CaHV core protein, or host response to the virus [14,15,16], and some information about hosts immune to CaHV have also been obtained [17]. Furthermore, the viral protein targets for mitochondria FoF1-ATPase that might provide energy for virus replication were also reported [18]. In contrast, little is known about the key factor for virus–host interactions or whether RING finger proteins encoded by aquatic animal herpesviruses are involved in viral biological processes [19,20].

RING finger proteins are a large family of proteins containing a C3HC4-type RING domain (RD) (Cys–X2–Cys–X9–39–Cys–Xl–3–His–X2–3–Cys–X2–Cys–X4–48–Cys–X2–Cys; C is cysteine, H is histidine), widely involved in diverse aspects of biological processes of cellular organisms [21,22] and human–virus life cycles [23] with E3 ubiquitin ligase activity. E3 ubiquitin ligase is a member of an enzymatic cascade for protein ubiquitination, including E1 ubiquitin-activating enzyme and E2 ubiquitin-conjugating enzyme [24]. Regarding aquatic viruses, RING finger proteins are only reported in infectious spleen and kidney necrosis virus (ISKNV) [25] and white spot syndrome virus (WSSV). The latter functions in virus latency, replication, and host protein degradation [26]; however, limited information about the RING finger proteins is known in fish herpesvirus [9]. 

The trend in studying pathogenic viruses is to offer a scientific basis for prevention and control tactics [27]. Understanding virus pathogenicity, its molecular biological characteristics, and viral gene function will contribute to more effective prevention of viral diseases and ensure the health of the aquaculture industry [28]; therefore, in this study, five RING finger-containing proteins (39L, 52L, 131R, 136L, and 143R) encoded by CaHV were screened based on comparison with previously described consensus sequences (C3HC4). These genes were cloned and expressed, subcellular localizations were observed, and their functions in ubiquitination were evaluated, respectively. Our research will lay a foundation for the detailed understanding of the functions of these viral proteins, and the molecular mechanism of fish herpesvirus–host interaction.

## 2. Materials and Methods

### 2.1. Virus, Cell Lines, and Reagents

The virus suspension and purified CaHV were previously stored in our laboratory at −80 °C [29,30]. Its complete genome was sequenced by our laboratory [13]. CaHV was isolated from challenged crucian carps (*Carassius auratus*) and used for further DNA extraction assay. *Epithelioma papulosum cyprinid* (EPC) cells were maintained in Medium 199 supplemented with 10% fetal bovine serum (FBS) at 25 °C [31], and used for transfection and fluorescence observation. Human embryonic kidney (HEK293T) cells were cultured under an atmosphere of 5% CO_2_ (37 °C) in fresh Dulbecco’s modified Eagle’s medium (DMEM) supplemented with 10% FBS [32], and used for transfection and ubiquitination detection. Ubiquitin, E1, and E2 enzymes were purchased from Boston Biochem. UbcH5a has been widely used and has proved to participate in ubiquitination more effectively with most identified E3 ubiquitin ligase proteins compared to other types of E2 [33]. Therefore, UbcH5a was chosen for our ubiquitination assays to identify the E3 activity of these RING finger proteins.

### 2.2. Sequence Analysis of RING Finger Protein Homologs

Five RING family genes were BLAST in the CaHV genome (Genbank accession: KU199244) [13], which encodes five RING finger proteins: 39L, 52L, 131R, 136L, and 143R. Their homologs were also BLAST and predicted in other fish herpesviruses according to their genome sequence. Then, the genes were labeled in their respective genomes and compared. The sequences are illustrated via Genbank accession numbers, SY-C1 (KM200722), ST-J1 (JQ815364), CyHV2-SY (KT387800), YZ-01 (MK260012), CNDF-TB2015 (MN201961), KHV-U (DQ657948), CyHV3-Cavoy (MG925485), CyHV3-E (MG925486), CyHV3-FL (MG925487), GZ11-SC (MG925488), CyHV3-I (MG925489), CyHV3-M3 (MG925490), CyHV3-T (MG925491), and CyHV1 (JQ815363) [34,35,36]. Positions of conserved domains on the RING finger proteins were analyzed using the BLAST and SMART programs. Nuclear localization signals (NLSs) in 52L were predicted using the cNLS Mapper program. Further analysis of RING domains was performed by taking domain sequences from National Center for Biotechnology Information (NCBI) for multiple sequence alignment using ClastalX 1.83, followed by GENEDOC.

### 2.3. Polymerase Chain Reaction (PCR) and Plasmid Construction

CaHV genome DNA was extracted from purified viruses by methods described previously [14], and used as a template to amplify the five genes and their isolated or truncated fragments. Amplified products were cloned into different vectors to produce recombinant plasmids used for fluorescence observation, prokaryotic expression, and eukaryotic expression. Detailed information is as follows. Primers used and the information about recombinant plasmids are shown in Table 1.

To produce plasmids for transfection and fluorescence observation, *39L*, *131R*, *136L,* and *143R* were amplified with pairs of primers *39L*-F/R, *131R*-F/R, *136L*-F/R, and *143R*-F/R, respectively. The PCR reaction was performed in a volume of 50 µL containing 0.5 µL of Fastpfu polymerase (TransGen, Beijing, China), 10 µL of 5× PCR buffer, 1 µL of each primer (10 mM), 4 µL of dNTPs (2.5 mM), and 1 µL of purified DNA. PCR conditions were carried out as follows: pre-denaturation at 94 °C for 5 min; 35 cycles of denaturation at 94 °C for 20 s, annealing at 55 °C for 20 s, and extension at 72 °C for 1 min; followed by a final extension step of 72 °C for 5 min. The PCR products were analyzed by electrophoresis in 1% agarose gel. Since there is an intron in the DNA sequence of *52L* in the genome, an overlap extension PCR was employed, including two rounds of PCR. Briefly, two DNA fragments were obtained from the first round PCR with the primers of *52L*-F/*52L*-linkerR and *52L*-linkerF/*52L*-R. PCR products were purified and then used as a template for the second round of PCR with the primers of *52L*-F/R. Then, *52L* with the intron deletion was obtained. Five target fragments were 1389 bp, 1953 bp, 1353 bp, 702 bp, and 1812 bp, conforming to expectation as shown in Table 1. According to the manufacturer’s protocol, these fragments were purified using a Silica Bead DNA Gel Extraction Kit (Fermentas, MA, USA) according to the manufacturer’s protocol, digested with corresponding restriction enzymes, and inserted into pEGFP-N3 to produce recombinant plasmids pEGFP-*39L*, pEGFP-*52L*, pEGFP-*131R*, pEGFP-*136L,* and pEGFP-*143R*. 

RING domain-deleted mutants *39L*Δ_6–47_, *131R*Δ_12–43_, and *143R*Δ_7–51_ were amplified with primers *39L*Δ_6–47_-F/R, *131R*Δ_12–43_-F/R, and *143R*Δ_7–51_-F/R, respectively, and subsequently cloned into plasmid pEGFP-N3 to construct recombinant plasmids pEGFP-*39L*Δ_6–47_, pEGFP-*131R*Δ_12–43_, and pEGFP-*143R*Δ_7–51_. *52L*Δ_565–607_-N and *52L*Δ_565–607_-C were obtained from the first round of PCR with the primers of *52L*Δ_565–607_-F/*52L*Δ_565–607_-R1 and *52L*Δ_565–607_-F1/*52L*Δ_565–607_-R, then purified and used as a template for the second round of PCR with the primers of *52L*Δ_565–607_-F/R to get the fragment of *52L*Δ_565–607_. *136L*Δ_45–87_ were obtained using the same method with primers of *136L*Δ_45–87_-F/*136L*Δ_45–87_-R1, *136L*Δ_45–87_-F1/*136L*Δ_45–87_-R, and *136L*Δ_45–87_-F/R. Then, they were subsequently cloned into plasmid pEGFP-N3 to construct recombinant plasmids pEGFP-*52L*Δ_565–607_ and pEGFP-*136L*Δ_45–87_.

Isolated RING domain coding fragments (*39L*_6–47_, *52L*_565–607_, *131R*_12–43_, *136L*_45–87_, *143R*_7–51_) were amplified with primers *39L*_6–47_-F/R, *52L*_565–607_-F/R, *131R*_12–43_-F/R, *136L*_45–87_-F/R, and *143R*_7–51_-F/R, respectively, and subsequently cloned into plasmid pEGFP-N3 to construct recombinant plasmids pEGFP-*39L*_6–47_, pEGFP-*52L*_565–607_, pEGFP-*131R*_12–43_, pEGFP-*136L*_45–87_, and pEGFP-*143R*_7–51_.

To produce plasmids for ubiquitination detection in cell culture, the five genes were amplified with primers *39L*-C-F/R, *52L*-C-F/R, *131R*-C-F/R, *136L*-C-F/R, and *143R*-C-F/R and inserted into pCMV-Tag2A, respectively, to construct recombinant plasmids pCMV-*39L*, pCMV-*52L*, pCMV-*131R*, pCMV-*136L,* and pCMV-*143R*. The cDNA fragment encoding ubiquitin (Ub) (GenBank accession no. M26880.1) was amplified with primers *Ub*-F/*Ub*-His-R by RT-PCR from the total RNA of HEK293T cells as described previously [37], and then cloned into the pcDNA3.1(+) to construct plasmid pcDNA3.1-*Ub*-His.

To construct plasmids for prokaryotic expression, two full-length genes (*131R* and *136L*) were amplified with primers *131R*-E-F/R, *136L*-E-F/R, respectively, their isolated RING domain coding fragments (*131R*_12–43_ and *136L*_45–87_) were amplified with primers *131R*_12–43_-E-F/R and *136L*_45–87_-E-F/R, and their RING domain-deleted mutants (*131R*Δ_12–43_ and *136L*Δ_45–87_) were amplified using pEGFP-*131R*Δ_12–43_ and pEGFP-*136L*Δ_45–87_ as a template with primers *131R*Δ_12–43_-E-F/R and *136L*Δ_45–87_-E-F/R, respectively. Subsequently, the obtained fragments were cloned into plasmid pET32a to construct recombinant plasmids pET*131R*, pET*136L,* pET*131R*_12–43_, pET*136L*_45–87_, pET*131R*Δ_12–43_, and pET*136L*Δ_45–87_. 

### 2.4. Transfection and Fluorescence Observation

EPC cells were inoculated on a microscopic coverslip in 6-well plates grown to 90% confluence. Then, 1.25 μg of each recombinant plasmid were transfected into EPC cells using the lipofectamine 3000 (Invitrogen, MA, USA) reagent (according to the manufacturer’s instruction). At 24 h post-transfection (hpt), cells were fixed with 4% paraformaldehyde (PFA) for 30 min, permeabilized with 0.2% Triton X-100 for 15 min, and stained with Hoechst 33342 (Sigma, MO, USA) for 15 min. The cells were observed under a Leica DMIRB fluorescence microscope (objective 100×), as described previously [16].

### 2.5. Ubiquitination Detection in Cell Culture

Ubiquitination assays were performed in HEK293T cells to detect whether these five RING finger proteins could cause ubiquitination. HEK293T cells were inoculated in 6-well plates grown to 90 % confluence. Each recombinant plasmid (1.25 μg) plus pcDNA3.1-*Ub*-His (1.25 μg) were transfected into HEK293T cells, respectively, using the lipofectamine 3000 reagent as mentioned above, and an empty pCMV-Tag2A was used as control. For the protein stability experiment, HEK293T cells were treated with the proteasome inhibitor MG132 (20 μM) at 28 hpt for 8 h. Then cells were harvested for immunoprecipitation at 36 hpt. Harvested HEK293T cells were washed with pre-cooled PBS three times, then lysed with 200 μL lysis buffer (20 mM Tris, 150 mM NaCl, 1% Triton X-100, pH 7.5) (Byotime, Shanghai, China) containing 2 μL protease inhibitor cocktail (Sigma) and 2 μL Phenylmethylsulfonyl Fluoride (PMSF) (100 mM). The mixtures were inverted at 4 °C for 1 h, and centrifuged at 15,493× *g* for 5 min at 4 °C. The supernatant was transferred to a new tube, followed by adding 10 μL of Anti-Flag Affinity Gel (Sigma) and gently inverted at 4 °C overnight. The immuno-precipitates were washed three times with pre-cooled PBS, and finally resuspended by 30 μL PBS. Then, 7 μL 5× loading buffers were added to each sample, boiled at 100 °C for 10 min, and subjected to 8% and 12% sodium dodecyl sulfate polyacrylamide gel electrophoresis (SDS-PAGE). Proteins were transferred to a PVDF membrane (Millipore) and detected by western blot after sequential incubation with the primary antibodies (anti-Ub and anti-His) against the indicated target proteins and corresponding horseradish peroxidase (HRP)-conjugated secondary antibodies. The membranes were incubated with an enhanced chemiluminescence reagent (Millipore). The chemiluminescent signals were detected using imager Tanon 5200. If a high molecular smear on the membrane was detected using an anti-Ub antibody, it would indicate that the corresponding product has ubiquitination catalyzing activity. The relative levels of ubiquitination were calculated as ratios to the corresponding protein normalization using Image J software. High values indicate high activities.

### 2.6. Prokaryotic Expression and Purification of Proteins

Recombinant plasmids were transformed into *E. coli* BL21 (DE3) competent cells. The positive clones were cultured in an Luria-Bertani (LB) medium with 0.1 mg/mL ampicillin to mid-log phase at 37 °C and then induced with 0.1 mM Isopropyl-beta-D-thiogalactopyranoside (IPTG) for 4 h at 24 °C. Bacterial pellets were resuspended in a 1× binding buffer (300 mM NaCl, 50 mM sodium phosphate buffer, 10 mM imidazole, pH 8.0), lysed by sonication on ice, and clarified by centrifugation at 16,000× *g* for 20 min. The supernatant containing recombinant proteins were then purified by Ni-NTA beads according to the manufacturer’s protocol with slight modifications as previously reported [38,39]. Un-induced bacteria, induced bacteria, and purified proteins were then verified on 12% SDS-PAGE by Coomassie blue staining. Purified proteins were dialyzed against ice-cold 50 mM Tris-HCl (pH 7.5). Protein concentrations were determined using an Enhanced BCA Protein Assay Kit according to the manufacturer’s instructions (Beyotime), adjusted to 0.4 μg/μL, respectively, and stored at −80 °C.

### 2.7. Ubiquitination Assays of Purified Proteins

To detect whether these five proteins have E3 ubiquitin ligase activity or not, purified proteins were used for ubiquitination assays, respectively. Reactions were performed as follows: 5 μL of mixtures (50 mM Tris-HCl, 5 mM MgCl_2_, 2 mM ATP, 50 nM E1 enzyme, 250 nM E2 enzyme, 5μg ubiquitin, pH 7.5) were mixed with 5 μL (2 μg) of each purified protein (131R, 136L, 131R_12–43_, 136L_45–87_, 131RΔ_12–43_, and 136LΔ_45–87_), respectively, to final volumes of 10 μL, and then incubated at 37 °C for 1 h. Each reaction was quenched with 5× loading buffer, boiled for 10 min, and subjected to electrophoresis on 8% or 12% SDS-PAGE, then analyzed by western blot with anti-Ub and anti-Flag antibodies as described in Section 2.5.

## 3. Results

### 3.1. Fish Herpesvirus Encoding RING Finger Proteins

**Figure 1:**
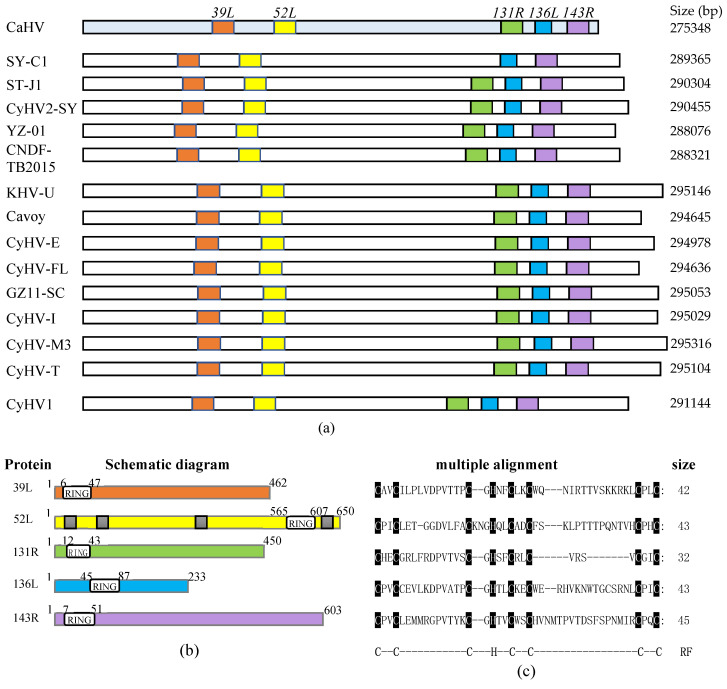
RING family genes layout in genome and sequences and structural characteristics of five proteins. (**a**) The genomes are shown to scale as horizontal bars. Colored squares indicate RING family genes in the Carassius auratus herpesvirus (CaHV) genome and their homologs in other fish herpesvirus genomes. (**b**) Schematic diagrams. White boxes, RING domain; Gray boxes in 52L, predicted nuclear localization signals (NLSs). (**c**) The multiple alignments of RING domains. The conserved amino acid residues in RING domains were covered by black shade. RF: RING domain. C, cysteine; H, histidine.

As shown in Figure 1a, five RING family genes (39L, 52L, 131R, 136L, and 143R) were screened in the CaHV genome. Further analysis showed that homologs of these five genes also exist in 14 other known fish herpesvirus genomes, with similar arrangements and number, except for SY-C1, which lacks the homolog of CaHV-*52L*. This indicates that RING family genes widely exist in fish herpesvirus, and probably play vital roles in virus infection.

Structural characteristics of the five proteins are shown in Figure 1b, and the lengths of these proteins were 462, 650, 450, 233, and 603 amino acids, respectively. All of them contain the RING domain. The sizes and positions of the domains are different, four of which are located at the N-terminal, and only 52L is located at the C-terminal. Besides, four nuclear localization signals (NLSs) were predicted in 52L. Multiple alignments are shown in Figure 1c. Sizes of RING domains were 42, 43, 32, 43, and 45 amino acids, respectively, containing conserved C3HC4 residues, which conformed to the classical RING domain.

### 3.2. Subcellular Localization of RING Finger Proteins

**Figure 2:**
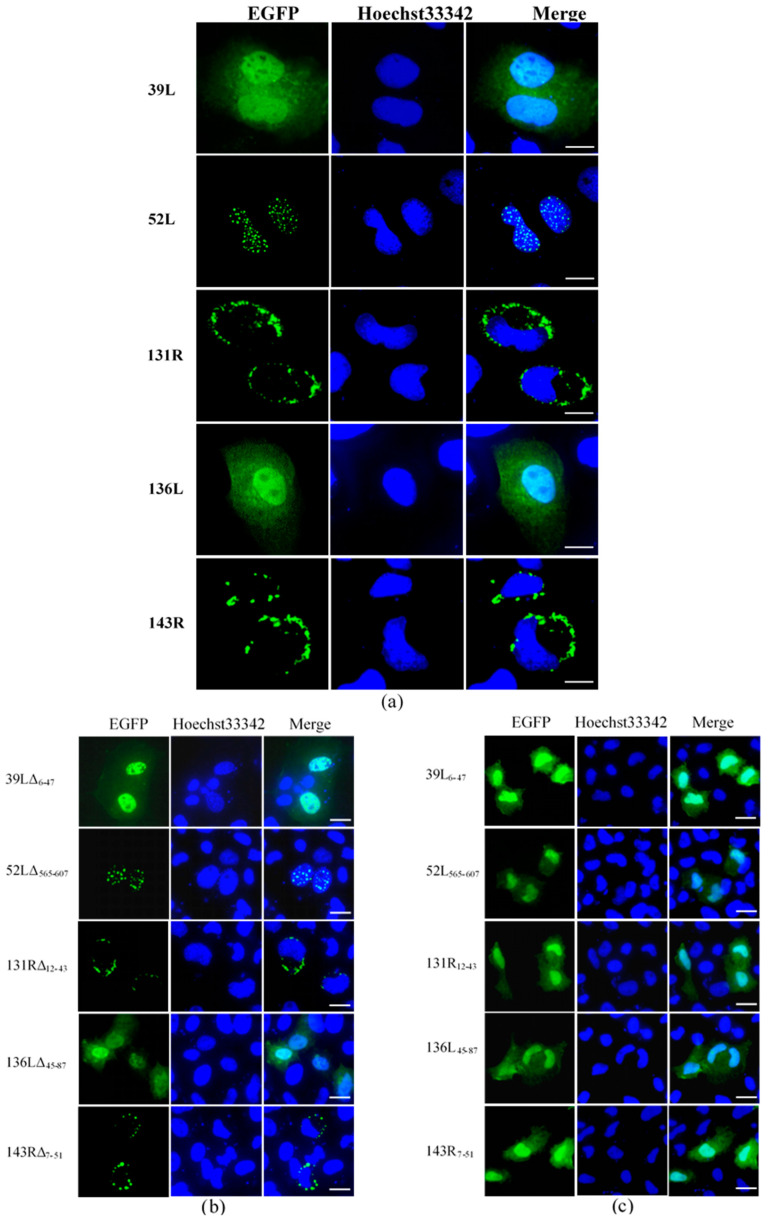
Fluorescence micrographs. (**a**) Five RING finger proteins (39L, 52L, 131R, 136L, and 143R). 39L and 136L diffused in the whole cell. 52L signaled punctate in the nucleus. 131R and 143R localized on cytomembrane. (**b**) RING domain-deleted mutants. The mutants showed similar localizations with corresponding full-length proteins. (**c**) RING domain isolates. The isolates are diffused in the whole cell. Bar = 10 μm.

Subcellular localization was determined by detecting the fluorescence distribution of the EGFP fusion proteins. As shown in Figure 2a, 39L and 136L diffused in the whole cell. 52L localized speckled at the nucleus. Both 131R and 143R localized on cytomembrane. After the RING domain deletion, 39LΔ_6–47_ and 136LΔ_45–87_ diffused in the whole cell, 52LΔ_565–607_ localized at the nucleus, 131RΔ_12–43_ and 143RΔ_7–51_ localized on cytomembrane, similar to their corresponding wild-type proteins (Figure 2b). This indicates that no effects of deleting RING domains on subcellular localization were detected. The isolates 39L_6–47_, 52L_565–607_, 131R_12–43_, 136L_45–87_, and 143R_7–51_ all diffused in the whole cell (Figure 2c), implying that the RING domain does not determine the subcellular localization here. The results above indicate that five RING finger proteins present different subcellular localization, determined by the sequences outside the RING domain.

### 3.3. Ubiquitination Activity of RING Finger Proteins in Cell Culture

**Figure 3:**
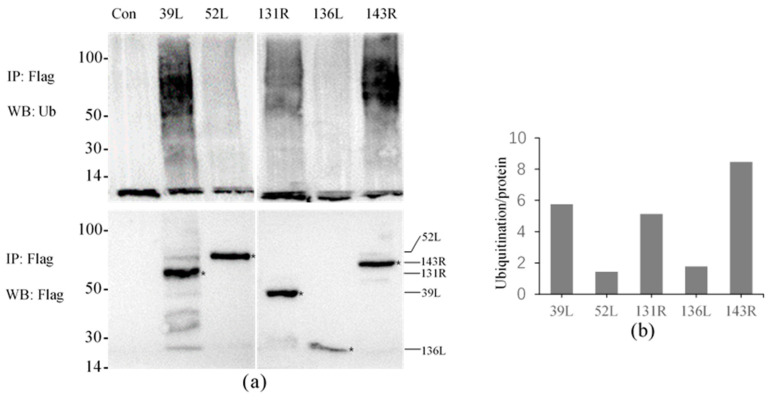
Ubiquitination activity of five RING finger proteins 39L, 52L, 131R, 136L, and 143R in cell culture. (**a**) Ubiquiti nation detection. The upper part shows ubiquitination detection, and the below expresses immunoprecipitated proteins detection. IP: Immunoprecipitation; WB: Western blot; Ub: ubiquitin. (**b**) Graph of the relative ubiquitination values. The values of 39L and 131R were approximately 5–6, 52L and 136L approximately 1–2, and 143R approximately 8–9.

The results showed that high molecular weight smears were detected in all samples except for the control using the anti-Ub antibody, and the molecular weights were mainly concentrated in 50–100 kDa (Figure 3a). This indicates that all five proteins presented ubiquitination activity, though the displayed activities were different. Proteins were obtained by co-IP, and detected molecular weights were approximately 51, 72, 49, 27, and 66 kDa by anti-Flag antibody, confirming that the Flag-tagged fusion proteins were effectively expressed and immunoprecipitated. 

The relative activities were compared by analyzing the intensity of the ubiquitination smear. As shown in Figure 3b, the relative activity values of 52L and 136L were only 1–2, other proteins about 5–9, and 143R showed the highest value about 8–9, which indicates its highest activity.

### 3.4. Identification of Prokaryotic Expression Products

**Figure 4:**
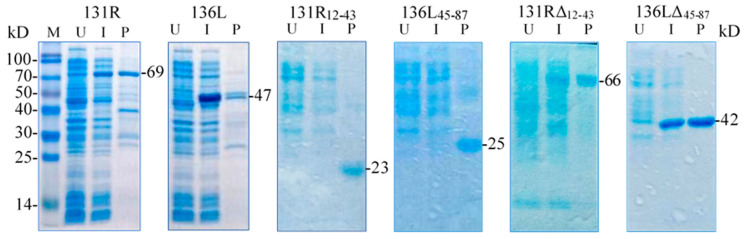
Sodium dodecyl sulfate polyacrylamide gel electrophoresis (SDS-PAGE) detected prokaryotic expression products of two recombinant full-length proteins 131R and 136L, two recombinant isolates 131R_12–43_ and 136L_45–87_, and two recombinant mutants 131RΔ_12–43_ and 136LΔ_45–87_. M: Protein marker; U: Un-induced bacteria; I: Induced bacteria; P: Purified protein. The recombinant proteins are indicated with short lines, and predicted molecular weights are shown on the right.

131R and 136L, their RING domain isolates 131R_12–43_ and 136L_45–87,_ and their RING domain-deleted mutants 131RΔ_12–43_ and 136LΔ_45–87_ were expressed and purified. As shown in Figure 4, compared to the lanes of un-induced products, specific bands were detected in lanes of induced and purified products, respectively. The detected protein molecular weights were approximately 69, 47, 23, 25, 66, and 42 kDa, which conformed to the predicted molecular weights, implying that the recombinant proteins were expressed and purified. Note the recombinant proteins were increased in size due to an approximately 21 kDa fused Trx-His-S-His tag from the vector pET32a. The purified proteins were then used in the ubiquitination assays to detect ubiquitination activity.

### 3.5. Ubiquitination Activity of the Recombinant Proteins

**Figure 5:**
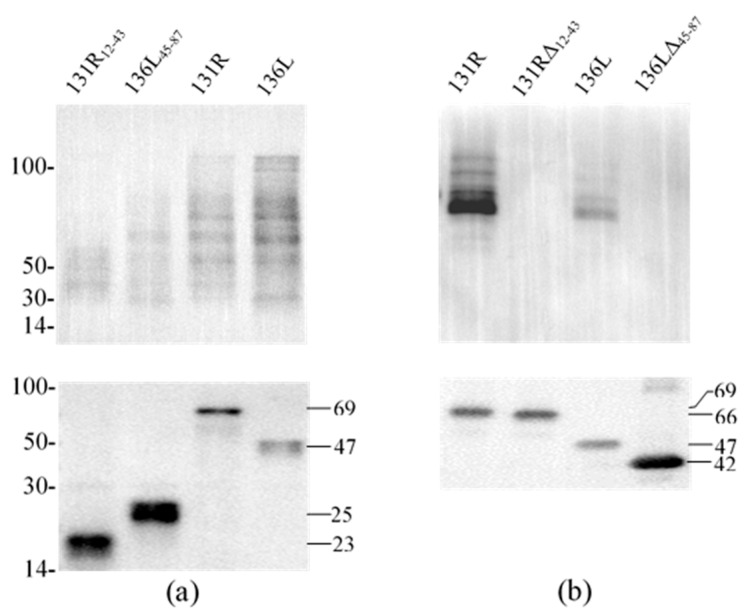
Western blot analysis for ubiquitination reaction products of purified proteins. Each subfigure contains two parts, detection of target proteins using anti-His antibody and detection of ubiquitination using anti-Ub antibody by western blot. (**a**) Ubiquitination reaction products of purified proteins (131R_12–43_, 136L_45–87_, 131R, and 136L) were detected using anti-Ub and anti-His, respectively. (**b**) Ubiquitination reaction products of purified proteins (131R, 131RΔ_12–43_, 136L, and 136LΔ_45–87_) were detected using anti-Ub and anti-His, respectively. WB: Western blot; Ub: ubiquitin.

To reveal the effects of the RING domain on ubiquitination activity, ubiquitination catalyzed by full-length proteins (131R and 136L), RING domain isolates (131R_12–43_ and 136L_45–87_), and RING domain-deleted mutants (131RΔ_12–43_ and 136LΔ_45–87_) were further compared. Recombinant 131R_12–43_ (23 kDa), 136L_45–87_ (25 kDa), 131R (69 kDa), 136L (47 kDa), 131RΔ_12–43_ (66 kDa), and 136LΔ_45–87_ (42 kDa) were detected by anti-His antibody (Figure 5a,b). Compared to full 131R and 136L, ubiquitination formed bands were detected fewer and weaker by anti-Ub in 131R_12–43_ and 136L_45–87_ catalyzing products, but not detected in 131RΔ_12–43_ and 136LΔ_45–87_. 

To confirm the precise activity of RING finger proteins, we performed ubiquitination assays in the incomplete reaction mixtures. Ubiquitinylated smears could be detected after respectively incubating 131R and 136L in the complete reaction mixtures. In the incomplete reaction mixtures lacking Mg^2+^, ATP, Ub, E1, E2, and purified RING finger proteins, no ubiquitination smear was detected. Because the complete reaction buffers used in the assays were free of any E3 enzymes other than the purified proteins, each recombinant protein functions as an E3 ubiquitin ligase, which confirmed their activity as an E3 ubiquitin ligase activity. Furthermore, in the incomplete reaction mixtures containing recombinant protein but lacking Mg^2+^, ATP, Ub, E1, or E2, no bands formed by ubiquitination were detected, indicating the necessity of these components for E3 ubiquitin ligase activities of these proteins [40]. This indicates that the RING finger proteins have E3 ligase activity, determined by the RING domain and influenced by the sequences outside the domain.

## 4. Discussion

In this study, multiple RING family genes were found to exist in the 15 known fish herpesviruses that were first reported; however, their relative locations in the genomes were uneven, and the sizes of the sequences among the homologs were also different. RING family genes were also predicted in other fish herpesvirus genomes, such as Anguillid herpesvirus 1 (AngHV-1), indicating that they widely exist in fish herpesviruses [41]. Whether these divergences related to gene expression or functions in virus-host interaction or infection needs further study. Genes layout, protein structures, subcellular localization, and ubiquitination activities were analyzed through bioinformatic analysis, fluorescence observation, and ubiquitination assay as shown in Table 2. Many questions need further study. As far as we know, this is the first report describing the ubiquitination and activity of RING finger proteins in fish herpesvirus.

This study indicates that RING finger proteins possess E3 ligase activity, and the RING domain determined the identified activity because the domain deletion abrogated the activity, as previously reported [42,43]. In addition, this study showed that the RING finger proteins catalyze ubiquitination in collaboration with E1 ubiquitin-activating enzymes and E2 ubiquitin-conjugating enzymes [44,45,46], and some viral RING finger proteins were directly or indirectly identified to interact with cellular E2 enzymes to mediate ubiquitination, providing evidence that these proteins probably hijack the host ubiquitination machine to function [47,48,49]. Here, CaHV encodes five RING finger proteins that can catalyze ubiquitination in complete reactions outside the cell. Incomplete reactions containing these proteins but lacking neither E1 nor E2 enzymes can catalyze ubiquitination. These proteins were able to catalyze ubiquitination, but no potential E1 and E2 enzymes were predicted in the CaHV genome that could participate in this process, meaning that these virus proteins hijacked cellular E1 and E2 enzymes to complete this process. This suggests that fish herpesvirus showed remarkable abilities in exploiting their hosts. Fish herpesviruses, especially cyprinid herpesviruses, were reported to infect fish that are not their specific host, even hybrids [50], which may connect to the similar cellular E1 E2 that also exist in the nonspecific host and hybrids.

Subcellular localization can provide clues for protein biological function [51], and different subcellular localizations suggest different functions. As previously reported, viral proteins such as ICP0 encoded by HSV-1 and core protein 91R encoded by *Rana grylio* virus (RGV) were localized at the nucleus, which has a nuclear localization signal (NLS), and play an important role in promoting the successful onset of lytic infection and productive reactivation of viral genomes from latency or virus genome replication [32,52,53,54]. Here, 52L of CaHV were observed localized at the nucleus and predicted containing an NLS, and probably have similar functions. Proteins localized in cytoplasm or whole cells might relate to virus incorporation, maturation or release, and sometimes targets viral replication factories, which might also involve virus replication and processing [55,56,57,58,59]. 39L and 136L showed similar locations here. Furthermore, those localized at membrane proteins were mostly related to viral entry or release, whereas viral membrane E3 ubiquitin ligase usually regulates cellular protein traffic, which probably helps virus maintenance, representing the potential functions of 131R and 143R [60,61]. As our results have shown, three types of subcellular localization among five proteins were observed. Various subcellular localization probably suggests that they perform functions in the different stages of virus infection.

## 5. Conclusions

In this study, we identified that the RING finger proteins exist widely in fish herpesviruses and that protein structure affects subcellular localization, relative to the level of activity, except for the RING domain, which determines the E3 ubiquitin ligase activity. The five virus RING finger proteins can exploit cellular E1 and E2 to complete the procedure of ubiquitination, which shows that the RING finger proteins directly participate in virus–host interaction. This indicates the multiparameter diversity of fish herpesvirus (CaHV) RING finger proteins. These results regarding fish herpesvirus encoded RING finger proteins may help us to further understand the interactions between virus and host.

## Figures and Tables

**Table 1:** Primers to amplify DNA and construct plasmids for localization, prokaryotic expression, and eukaryotic expression.

Primers	Primer Sequence (5′ to 3′)	Plasmids	Size of DNA Fragments (bp)
*39L*-F	AACAAGCTTAATAATGGAAGACACG (HindIII)	pEGFP-*39L*	1389
*39L*-R	GATGGTACCGACATGGTTCGAC (KpnI)		
*52L*-F	AGAAAGCTTAACACGATGCCGAC (HindIII)	pEGFP-*52L*	1953
*51L*-R	TCTGAGGTACCACATGTGACCATAG (KpnI)		
*52L*-linkerF	AGGCCTCAACAGGTGCCCGATACC		
*52L*-linkerR	GGTATCGGGCACCTGTTGAGGCCT		
*131R*-F	ACAAAGCTTCAACCATGGATCGT (HindIII)	pEGFP-*131R*	1353
*131R*-R	AATCCGGATCCGAGTGTTAGAGT (BamHI)		
*136L*-F	TTTGAATTCGACAATGTCCACCT (EcoRI)	pEGFP-*136L*	702
*136L*-R	CGCGGATCCGAGTATGCTGCAGAAGC (BamHI)		
*143R*-F	CAGCAGAAGCTTCTAAAAATGACG (HindIII)	pEGFP-*143R*	1812
*143R*-R	CACAGGATCCGATATTAGCTACAAT (BamHI)		
*39L*Δ_6–47_-F	TCAGATCTCGAGCTCAAGCTTATGGAAGACACGGACAGAGAAAAGGTGAAGGGCACG (HindIII)	pEGFP-*39L*Δ_6–47_	1263
*39L*Δ_6–47_-R	GGATCCCGGGCCCGCGGTACCGACATGGTTCGACGTGACAA (KpnI)		
*52L*Δ_565–607_-F	TCAGATCTCGAGCTCAAGCTTATGCCGACCTGGCCCATGTTT (HindIII)	pEGFP-*52L*Δ_565–607_	1824
*52L*Δ_565–607_-R	GGATCCCGGGCCCGCGGTACCACATGTGACCATAGACTTAAAGG (KpnI)		
*52L*Δ_565–607_-F1	GCTACCGTTCTCGGA GACGTTGCCCTGGTCAATTAT		
*52L*Δ_565–607_-R1	GACCAGGGCAACGTC TCCGAGAACGGTAGCCACTTC		
*131R*Δ_12–43_-F	TCAGATCTCGAGCTCAAGCTTATGGATCGTGAGACTCTACTCGGGCACCTGAGCCTGATAGAGTGCAGTCAACCGT (HindIII)	pEGFP-*131R*Δ_12–43_	1257
*131R*Δ_12–43_-R	GGATCCCGGGCCCGCGGTACCGAGTGTTAGAGTTATGGAAGC (KpnI)		
*136L*Δ_45–87_-F	TCAGATCTCGAGCTCAAGCTTATGTCCACCTACGTTGATATG (HindIII)	pEGFP-*136L*Δ_45–87_	573
*136L*Δ_45–87_-R	GGATCCCGGGCCCGCGGTACCGAGTATGCTGCAGAAGC (KpnI)		
*136L*Δ_45–87_-F1	GTCAAGTCGTTGATG AACAGGGACGTACCCGATAAT		
*136L*Δ_45–87_-R1	GGGTACGTCCCTGTT CATCAACGACTTGACAAAGTTG		
*143R*Δ_7–51_-F	TCAGATCTCGAGCTCAAGCTTATGACGGAGCCTCTGGATAGGACCATGGTGTGCAAAAAC (HindIII)	pEGFP-*143R*Δ_7–51_	1677
*143R*Δ_7–51_-R	GGATCCCGGGCCCGCGGTACCGATATTAGCTACAATAGTGGC (KpnI)		
*39L*_6–47_-F	TCAGATCTCGAGCTCAAGCTTATGGAAGACACGGACTGTGC (HindIII)	pEGFP-*39L*_6–47_	126
*39L*_6–47_-R	GGATCCCGGGCCCGCGGTACCTCTGAGCGTGCCCTTCAC (KpnI)		
*52L*_565–607_-F	TCAGATCTCGAGCTCAAGCTTCCGTTCTCGGATGTCCCAT (HindIII)	pEGFP-*52L*_565–607_	129
*52L*_565–607_-R	GGATCCCGGGCCCGCGGTACCATAATTGACCAGGGCAACG (KpnI)		
*131R*_12–43_-F	TCAGATCTCGAGCTCAAGCTTTGCCACGAGTGCGGCAGAC (HindIII)	pEGFP-*131R*_12–43_	96
*131R*_12–43_-R	GGATCCCGGGCCCGCGGTACCACAGATTCCACACACCGATCTG (KpnI)		
*136L*_45–87_-F	TCAGATCTCGAGCTCAAGCTTTGTCCCGTGTGCTGCGAG (HindIII)	pEGFP-*136L*_45–87_	129
*136L*_45–87_-R	GGATCCCGGGCCCGCGGTACCACAGATGGGACACAGGTTACG (KpnI)		
*143R*_7–51_-F	TCAGATCTCGAGCTCAAGCTTTGTCCCGTGTGCCTGGAAATG (HindIII)	pEGFP-*143R*_7–51_	135
*143R*_7–51_-R	GGATCCCGGGCCCGCGGTACCACACTGTGGGCACCTGATC (KpnI)		
*131R*-E-F	ACAGGATCCATGGATCGTGAGACTCTACTC (BamHI)	pET*131R*	1353
*131R*-E-R	TCCAAGCTTGAGTGTTAGAGTTATGGAAGC (HindIII)		
*136L*-E-F	CGCGGATCCATGTCCACCTACGTTGATATG (BamHI)	pET*136L*	702
*136L*-E-R	CCGCTCGAGGAGTATGCTGCAGAAGC (XhoI)		
*131R*_12–43_-E-F	GCCATGGCTGATATCGGATCCTGCCACGAGTGCGGCAGAC (BamHI)	pET*131R*_12–43_	96
*131R*_12–43_-E-R	CTCGAGTGCGGCCGCAAGCTTACAGATTCCACACACCGATCTG (HindIII)		
*136L*_45–87_-E-F	GCCATGGCTGATATCGGATCCTGTCCCGTGTGCTGCGAG (BamHI)	pET*136L*_45–87_	129
*136L*_45–87_-E-R	CTCGAGTGCGGCCGCAAGCTTACAGATGGGACACAGGTTACG (HindIII)		
*131R*Δ_12–43_-E-F	ATCGGATCCGAATTCGAGCTCATGGATCGTGAGACTCTACTC (SacI)	pET*131R*Δ12–43	1257
*131R*Δ_12–43_-E-R	CTCGAGTGCGGCCGCAAGCTTGAGTGTTAGAGTTATGGAAGC (HindIII)		
*136L*Δ_45–87_-E-F	ATCGGATCCGAATTCGAGCTCATGTCCACCTACGTTGATATG (SacI)	pET*136L*Δ45–87	573
*136L*Δ_45–87_-E-R	CTCGAGTGCGGCCGCAAGCTTGAGTATGCTGCAGAAGC (HindIII)		
*39L*-C-F	CCGGAATTCGATGGAAGACACGGACTGTGC (EcoRI)	pCMV-*39L*	1389
*39L*-C-R	CCCAAGCTTGACATGGTTCGACGTGACAA (HindIII)		
*52L*-C-F	CCCAAGCTTGATGCCGACCTGGCCCATGTTT (HindIII)	pCMV-*52L*	1953
*52L*-C-R	ACGCGTCGACCTAACATGTGACCATAGACTT (SalI)		
*131R*-C-F	CGCGGATCCGATGGATCGTGAGACTCTACTC (BamHI)	pCMV-*131R*	1353
*131R*-C-R	CCCAAGCTTGAGTGTTAGAGTTATGGAAGC (HindIII)		
*136L*-C-F	CGCGGATCCGATGTCCACCTACGTTGATATG (BamHI)	pCMV-*136L*	702
*136L*-C-R	CCGCTCGAGGAGTATGCTGCAGAAGC (XhoI)		
*143R*-C-F	CGCGGATCCGATGACGGAGCCTCTGGATTG (BamHI)	pCMV-*143R*	1812
*143R*-C-R	CCCAAGCTTGATATTAGCTACAATAGTGGC (HindIII)		
*Ub*-F	CCCAAGCTTATGCAGATCTTCGTGAAGACTC (HindIII)	pcDNA3.1-*Ub*-His	231
*Ub*-His-R	CGGGGTACCTTAATGGTGATGGTGATGATGCCCACCTCTGAGACGGAGC (KpnI)		

The restriction endonuclease sites are underlined. Numbers in the subscript are Really interesting new genes (RING) domain amino acids intercepted from protein sequences.

**Table 2:** Ubiquitination of full-length proteins, isolates, and mutants.

Open Reading Frames (ORFs)	Position of RING Domain	Remained Amino Acid Sizes	Subcellular Localization	Ubiquitination in Cell Culture	Ubiquitination of Purified Proteins
39L	N-terminal	1–462	Whole cell	++	nd
52L	C-terminal	1–650	Cellular nucleus	+	nd
131R	N-terminal	1–450	Cyto-plasmembrane	++	++
136L	N-terminal	1–233	Whole cell	+	++
143R	N-terminal	1–603	Cyto-plasmembrane	++	nd
Isolates					
131R_12–43_	Remain	12–43	Whole cell	nd	+
136L_45–87_	Remain	45–87	Whole cell	nd	+
Mutants					
131RΔ_12–43_	Deletion	1–11, 44–450	Cyto-plasmembrane	nd	−
136LΔ_45–87_	Deletion	1–44, 88–450	Whole cell	nd	−

Δ: deletion, −: none, +: weak, ++: strong, nd: not detected.

## Data Availability

Not applicable.
